# Efficacy of high-affinity liposomal astaxanthin on up-regulation of age-related markers induced by oxidative stress in human corneal epithelial cells

**DOI:** 10.3164/jcbn.18-27

**Published:** 2018-11-30

**Authors:** Tatsuharu Shimokawa, Mai Yoshida, Tatsuya Fukuta, Tamotsu Tanaka, Toshio Inagi, Kentaro Kogure

**Affiliations:** 1 Department of Pharmaceutical Health Chemistry, Institute of Biomedical Sciences, Tokushima University Graduate School, 1 Shomachi, Tokushima 770-8505, Japan; 2 Fuji Research Laboratories Pharmaceutical Division, Kowa Company, Ltd., 332-1 Ohnoshinden, Shizuoka 417-8650, Japan; 3 Kyoto Pharmaceutical University, Misasagi-Nakauchi-cho 5, Yamashina-ku, Kyoto 607-8414, Japan

**Keywords:** dry eye, desiccation stress, age-related markers, astaxanthin, liposomes

## Abstract

Decreases in tear volume, unstable tear films and excessive tear evaporation are known to cause desiccation and hyperosmolar stress. These, in turn, induce oxidative stress that is thought to cause dry eye, which is also considered to be age-related disease. We hypothesized that oxidative stress induces up-regulation of age-related markers, and that the antioxidant astaxanthin prepared as a liposomal formulation may be a candidate for the treatment of dry eye. Herein, we examined age-related markers in an *in vitro* dry eye model, and evaluated the efficacy of high-affinity liposomes containing astaxanthin. The *in vitro* dry eye model showed desiccation time-dependent increases in reactive oxygen species. We confirmed the up-regulation of p53, p21 and p16 as a function of desiccation time. Pretreatment with both neutral and slightly-positively-charged astaxanthin liposomal formulations showed significant suppression of up-regulation of all markers, with the positively-charged liposomes exhibiting the greatest efficacy. Furthermore, positively-charged liposomes labeled with fluorescent dyes demonstrated much higher affinity to normal human corneal epithelial cells (HCECs) than neutral liposomes. Taken together, we confirmed the up-regulation of age-related markers, especially p16, in an *in vitro* dry eye model, and demonstrated the potential of high-affinity liposomal astaxanthin for the treatment of dry eye.

## Introduction

Dry eye is a multifactorial disease characterized by a loss of homeostasis of the tear film, which is associated with tear film instability and hyperosmolarity, as well as inflammation and damage of the ocular surface.^(1)^ Dry eye is recognized as a common eye disease due to the large population of patients exhibiting dry eye worldwide, with a prevalence ranging from 0.4–33%, depending on environment and diagnosis criteria.^(2,3)^ Cyclosporine is currently used as the main treatment for dry eye worldwide, while other therapeutics, such as artificial eye drops, steroids, diquafosol sodium, rebamipide and lifitegrast are also used. However, development of more effective therapeutics is needed to prevent administration failure and decreased adherence resulting from long-term medication use. It is recognized that dry eye is caused by decreases in tear volume, unstable tear films and excessive tear evaporation, which leads to desiccation and hyperosmolar stress.^(1,4)^ These, in turn, induce oxidative stress caused by reactive oxygen species (ROS) derived from mitochondrial dysfunction^(5,6)^ induced by mitochondrial depolarization, resulting in inhibition of the mitochondrial respiratory chain. ROS contribute to DNA oxidation and inhibit gene transcription and DNA replication via the DNA damage response (DDR).^(6,7)^ In fact, several reports have shown that increases in mitochondrial damage, ROS generation and oxidative stress markers such as 8-hydryxy-2'-deoxyguanosine (8-OHdG), malondialdehyde (MDA) and 4-hydroxy-2-nonenal (4-HNE), are induced in both *in vitro* and *in vivo* dry eye models,^(6,8–10)^ suggesting that oxidative stress may be one possible cause of dry eye.

Dry eye is also considered to be an age-related disease, and a number of reports have shown that the prevalence of dry eye increases with age.^(2,11–13)^ These reports also suggest that women have a higher risk of developing dry eye disease than men, with the differences becoming significant with age. The aging process is normally explained by cell cycle arrest involved activation of the p53/p21 pathway, which inhibits cyclin E-cyclin-dependent kinase (Cdk) 2, and p16, which inhibit cyclin D-Cdk4 and cyclin D-Cdk6.^(14–16)^ These age-related markers are also known to be activated by a variety of endogenous and exogenous stressors. In particular, the p53/p21 pathway is stimulated by DDR, and ataxia telangiectasia mutated (ATM) or ataxia telangiectasia and Rad3-related (ATR) kinases inhibit cell-cycle progression via stabilization of p53 and transcriptional activation of p21 in this pathway.^(16)^

As aging is widely thought to be associated with oxidative stress, a possible cause of dry eye as noted above,^(17,18)^ we hypothesized that oxidative stress caused by desiccation stress may induce up-regulation of age-related markers, such as p21, p53 and p16. Antioxidants may therefore represent potential candidates for the treatment of dry eye via inhibition of DDR induced by ROS. To test this hypothesis, we first evaluated ROS and age-related markers in an *in vitro* dry eye model using normal human corneal epithelial cells (HCECs), and subsequently investigated the effect of an antioxidant in the model.

We chose astaxanthin (Asx, 3,3'-dihydroxy-β, β-carotene-4,4'-dione) as the antioxidant in this model. Asx is a carotenoid, and a common red pigment found in algae, fish and birds.^(19,20)^ Asx is widely used as a nutritional supplement and a cosmetic ingredient, and its safety has been demonstrated in humans. In addition, Asx has been reported to be more effective than other representative antioxidants, such as β-carotene and α-tochopherol, for prevention of singlet oxygen formation, as well as inhibition of lipid peroxidation in biological membranes.^(21–26)^ Moreover, as Asx exhibits high scavenging activity toward hydroxyl radicals, treatment with Asx is expected to prevent the pathogenesis of ROS-related diseases.^(26,27)^ However, as application of Asx for ocular administration requires use of aqueous eye drops, the high hydrophobicity of Asx may be problematic. Moreover, Asx developed as eye drops also has to overcome quite low bioavailability caused by the drainage system associated with tear flow from the lacrimal gland to the nasolacrimal duct.

In this study, we first clarified the relationship between ROS and age-related markers in an *in vitro* dry eye model using HCECs. To overcome the limitations associated with the ocular application of Asx noted above, we prepared liposomal formulations of Asx comprised of cationic lipids to allow for both homogenous dispersion in aqueous solution, and strong interactions with ocular cells via electrostatic effects, and subsequently evaluated the potential of the resultant liposomal formulations for treatment of dry eye.

## Materials and Methods

### Materials

Normal human corneal epithelial cells (HCECs) isolated from human corneal tissue and expanded twice, OcuLife Basal Medium, OcuLife Lifefactor that includes 15 ml of 200 mM l-glutamin, 0.5 ml of 5 mg/ml rh insulin, 0.5 ml of 1 mg/ml epinephrine bitartrate, 0.5 ml of 5 mg/ml apo transferrin, 2 ml of bovine pituitary extract, 0.5 ml of 100 µg/ml hydrocortisone hemisuccinate, 1 ml of OcuFactor and 0.5 ml of 30 mg/ml gentamicin and 15 µg/ml amphotericin B, a mixture solution of 0.05% trypsin and 0.02% ethylenediaminetetraacetic acid (EDTA), and phosphate buffered saline (PBS) containing 5% fetal bovine serum (FBS), all of which were purchased from Kurabo (Osaka, Japan). Transwell insert (24 mm diameter, 0.4 µm pore size) was obtained from Corning Incorporated (Acton, MA). Trypan blue was obtained from Wako Pure Chemical Industries (Osaka, Japan). Total ROS Detection Kit was purchased from Enzo Life Sciences (Lausen, Switzerland). RNeasy Plus Mini kit was obtained from Qiagen GmbH (Hilden, Germany). PrimeScript RT Master Mix (Perfect Real Time) and SYBR Premix Ex Taq II (Tli RNaseH Plus) were purchased from Takara Bio (Otsu, Japan). Egg phosphatidylcholine (EPC) was obtained from Sigma-Aldrich (St. Louis, MO). 1,2-Dioleoyloxy-3-3trimethylammonium propane chloride (DOTAP) was purchased from NOF Corporation (Tokyo, Japan). Astaxanthin was obtained from Wako Pure Chemical Industries (Osaka, Japan). Aminophenyl fluorescein (APF) was purchased from Goryo Chemical (Sapporo, Japan). 1,1'-Dioctadecyl-3,3,3',3'-tetramethylindocarbocyanine perchlorate (DiI) was obtained from Invitrogen (Carlsbad, CA). All other reagents were of the highest grade commercially available.

### Cell culture

Complete medium was prepared by adding OcuLife Lifefactor to OcuLife Basal Medium and mixing well. HCECs were cultured using complete medium in a 100 mm dish at 37°C in a humidified incubator with 5% CO_2_.

### *In vitro* dry eye model

The *in vitro* dry eye model was prepared as previous report^(28)^ with slight modifications. 7.5 × 10^4^ of HCECs were seeded onto the 24 mm insert of a Transwell 6 well plate, followed by addition of 1.4 ml and 2.6 ml of complete medium without hydrocortisone hemisuccinate to the upper and bottom layers, respectively. Cells were cultured for 48 h. After incubation, the Transwell plate containing HCECs was transferred to a clean bench and the medium was removed. Complete medium without hydrocortisone hemisuccinate (1.3 ml) was added to the bottom layer only. HCECs were desiccated uncovered in a clean bench for 0, 0.5, 1, and 2 h.

### Evaluation of cell viability in the *in vitro* dry eye model

HCECs were removed from the Transwell insert after each treatment using a solution of 0.05% trypsin and 0.02% EDTA, and neutralized by PBS containing 5% FBS, followed by staining using 0.3% trypan blue dissolved in PBS. The number of living HCECs was counted using a phase-contrast microscope (Olympus, Tokyo, Japan) and a hemocytometer (Hirschmann EM Techolor, Eberstadt, Germany). Cell viability was calculated as the ratio of the number of living HCECs following desiccation treatment to control cells without desiccation.

### Measurement of intracellular ROS after desiccation treatment

ROS production in HCECs after each desiccation treatment was determined using a Total ROS Detection Kit according to the manufacturer’s instructions. Following desiccation treatment, HCECs were washed with wash buffer and incubated with ROS detection solution provided by the manufacturer at 37°C for 30 min. HCECs were then removed from the Transwell insert in a manner similar to that of the cell viability test, and cell counting was performed. The cell density of each sample was normalized to PBS buffer, and the fluorescence intensities were analyzed using a microplate-reader (Tecan, Manchester, UK) operating at excitation and emission wavelength of 488 nm and 520 nm, respectively.

### RNA isolation from HCECs and evaluation of age-related markers using real-time PCR

Total RNA was extracted from desiccation-treated HCECs using an RNeasy Plus Mini kit according to the manufacturer’s instructions. The total RNA concentration was quantified using a Nanophotometer (Implen, Germany), followed by confirming the A260/280 and A260/230 ratios. Thereafter, cDNA was synthesized from 200 ng of total RNA using PrimeScript RT Master Mix (Perfect Real Time) and a MJ Mini Personal Thermal Cycler (BioRad Laboratories, Hercules, CA). The conditions for the reverse transcription (RT) reaction were 37°C for 15 min, while those for inactivation of reverse transcriptase were 85°C for 5 s. Real-time PCR was then performed using SYBR Premix Ex Taq II (Tli RNaseH Plus) and a Thermal Cycler Dice Real Time System III (Takara Bio). The amplification methods were as follows: initial denaturation at 95°C for 30 s, followed by 40 cycles of 95°C for 5 s and 60°C for 30 s for β-actin and p53, or 50 cycles of 95°C for 5 s and 65°C for 1 min for p16 and p21. The primer sequences used in this study are listed in Table 1. mRNA levels of p53, p21 and p16 were determined using the 2^−ΔΔCt^ method by normalizing to β-actin mRNA.

### Preparation of liposomal formulations of Asx

EPC or EPC/DOTAP liposomes both encapsulating Asx (E/Asx-lipo or E/D/Asx-lipo, respectively) were prepared using a lipid hydration method. Chloroform solution containing 20 mM of EPC or 18 mM of EPC and 2 mM of DOTAP, with or without 200 µM of Asx, was dried to a thin film using nitrogen gas in a test tube. Liposomal formulations that did not contain Asx (E-lipo and E/D-lipo, respectively) were also prepared as vehicles. Dried lipid films were hydrated with PBS at room temperature to obtain a liposomal suspension, and the particles were diminished in size by sonication using a bath type sonicator (Bransonic, Branson Ultrasonics Corporation, Danbury, CT). Finally, the liposomal suspensions were extruded using a Mini-Extruder (Avanti polar Lipids, Alabaster, AL) with polycarbonate membrane filters (pore size 0.2 µm, Whatman, Florham Park, NJ) to adjust the diameters of liposomes as well as to sterilize them.

### Determination of physicochemical properties of liposomes

The diameters and zeta potentials of the liposomal formulations were measured using a Zetasizer Nano (Malvern Instruments, UK). To analyze the concentration of Asx in each liposomal preparation, 40 µl of liposomal suspension was dissolved in a mixture of chloroform:methanol = 2:1, and the total volume was adjusted to 1 ml. Calibration curves samples were also prepared by adding a known amount of Asx and EPC or EPC and DOTAP to the chloroform:methanol solvent, and adjusting the volume to 1 ml. The absorbance values of the calibration curve samples and the liposomal samples were measured using a Nanophotometer, and the concentration of Asx in the liposomes was determined using the equation obtained via linear regression of the calibration curve.

### Measurement of hydroxyl radical production by Fenton reaction

Hydroxyl radical generation was evaluated by fluorescence intensity via Fenton raction. Hydroxyl radicals were generated by mixing the following in a test tube to yield a total volume of 300 µl: 60 µl of 100 µM APF (final concentration: 20 µM), 60 µl of an aqueous solution of 1 mM FeSO_4_ (final concentration: 200 µM) pH-adjusted to 4.5 using HCl, 0–45 µl of liposomal suspension (final Asx concentration: 0–30 µM), 75–120 µl of deionized distilled water, and 60 µl of 10 mM H_2_O_2_ (final concentration: 2 mM). One minute after mixing, fluorescence intensities were measured using a microplate-reader operating at excitation and emission wavelengths of 490 nm and 515 nm, respectively. Hydroxyl radical scavenging ratios were calculated as the fluorescence intensities of liposomes containing Asx to those without Asx.

### Pretreatment with liposomes in the *in vitro* dry eye model

To evaluate the efficacies of each liposomal formulation, HCECs prepared as described above were pretreated with either liposomal Asx (E/Asx-lipo or E/D/Asx-lipo) or vehicle (E-lipo or E/D-lipo) dispersed in complete medium without hydrocortisone hemisuccinate for 1 h just prior to desiccation treatment.

### Evaluation of association between liposomes with HCECs

E/Asx-lipo and E/D/Asx-lipo labeled with DiI as a fluorescence lipid dye were prepared using the methods described above. 1.5 × 10^5^ of HCECs were seeded onto a 35 mm dish and 2 ml of the complete medium without hydrocortisone hemisuccinate was added, followed by culturing for 48 h at 37°C in a humidified incubator with 5% CO_2_. After incubation, the medium was replaced with a new one containing each concentration of DiI-labeled liposomal Asx, and then cultivated for 1 h under the same conditions. After washing the HCECs 3× with 1 ml of PBS, 1 ml of new complete medium was added to the dish. Fluorescence images were obtained using a fluorescence microscope (Axio vert.A1, Carl Zeiss, Jena, Germany). The medium was removed from the dish and lysates were obtained by adding 200 µl of lysis buffer containing with PBS and 1% of *n*-octyl-β-d-glucoside. 100 µl of the supernatant of the centrifuged lysate was transferred to the microplate and the fluorescence intensities were measured using a microplate-reader operating at excitation and emission wavelength of 549 nm and 592 nm, respectively. Exposure levels of HCECs in each liposomal concentration were evaluated using fluorescence intensities.

### Statistical analysis

Data were expressed as means ± SD. Statistics analysis was performed by a paired student’s *t* test for comparison of two samples. *P*<0.05 was considered statistically significant.

## Results

### Influence of desiccation stress in the *in vitro* dry eye model

**Fig. 1:**
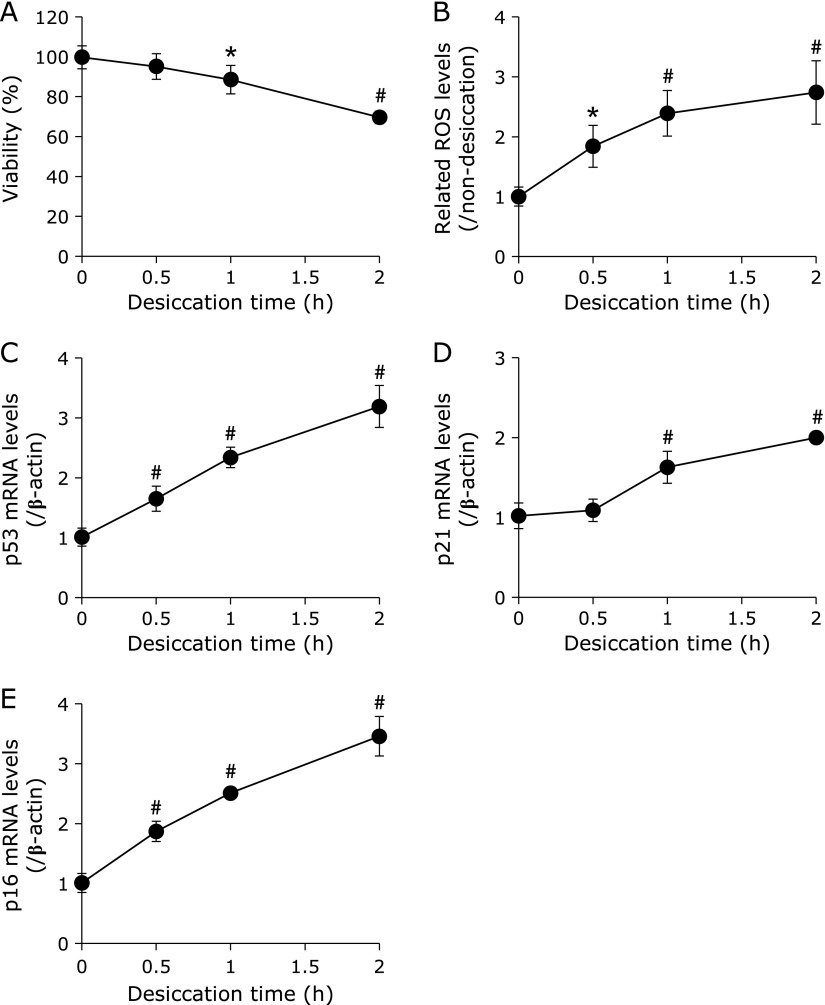
Time-dependence of each parameter in the *in vitro* dry eye model. Figures show results of cell viability (A), related intracellular ROS levels (B), mRNA levels of p53 (C), p21 (D) and p16 (E) after each desiccation time treatment in the *in vitro* dry eye model. Data are expressed as mean ± SD (*n* = 3–6). *p<0.05, ^#^*p*<0.01 vs desiccation treatment at 0 h.

Cell viability, intracellular ROS levels and the behaviors of age-related markers following desiccation treatment were examined in HCECs. Cell viability was significantly decreased after 1 h of desiccation treatment compared with no desiccation treatment (Fig. 1A). On the other hand, desiccation stress induced time-dependent production of intracellular ROS after 0.5–2 h of desiccation treatment (Fig. 1B). p53 mRNA levels were significantly up-regulated, even after 0.5 h of desiccation treatment, which continued through 2 h after treatment (Fig. 1C). Compared with p53, there was no significant difference in p21 mRNA levels at 0.5 h, but levels were significantly up-regulated after 1 h of desiccation treatment and continued through 2 h (Fig. 1D). p16 mRNA levels were also significantly up-regulated in a time-dependent manner from 0.5 to 2 h after desiccation treatment (Fig. 1E).

### Characteristics of liposomal formulations

**Fig. 2:**
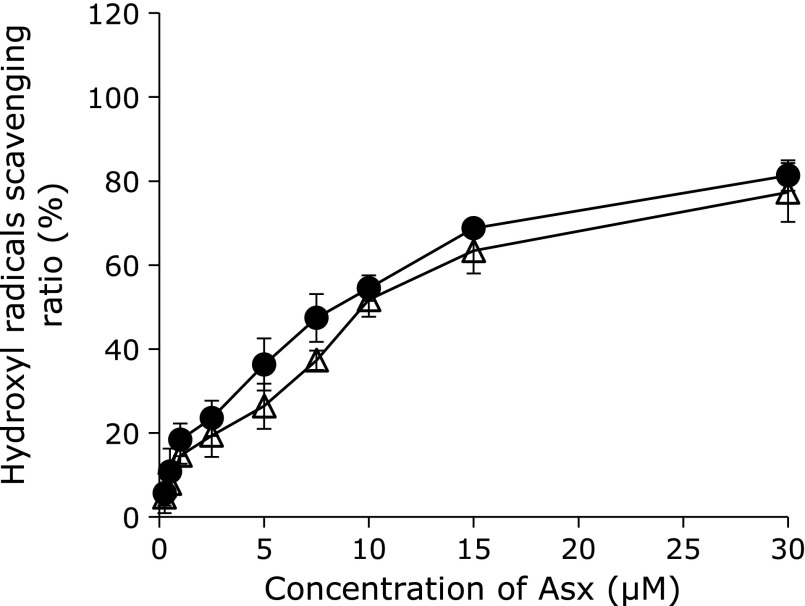
Hydroxyl radical scavenging curves of E/Asx-lipo and E/D/Asx-lipo in aqueous solution. Hydroxyl radical scavenging ability of each liposomal formulation was calculated as the ratio of E/Asx-lipo to E-lipo (●), or E/D/Asx-lipo to E/D-lipo (△). Data are expressed as mean ± SD (*n* = 3).

Before applying liposomes to the *in vitro* dry eye model, we first confirmed the physicochemical characteristics and hydroxyl radical scavenging ability of each liposomal formulation. The average diameters of all liposomes were approximately 140 nm (Table 2), which was thought to be small enough for use in the present study. The average polydispersity index (PDI) of all liposomes was approximately 0.325 (Table 2), with the data showing a single peak pattern indicative of similar particle size distributions. Regardless of whether Asx was encapsulated or not, results of zeta potential measurements showed a mostly neutral charge for liposomes comprised of EPC, and a slightly-positive charge for liposomes comprised of EPC and DOTAP, as expected (Table 2). Actual concentrations of Asx encapsulated in E/Asx-lipo and E/D/Asx-lipo were analyzed by absorptiometry and calculated to be close to the theoretical values, namely, 99.2% for E/Asx-lipo and 99.4% for E/D/Asx-lipo (Table 2). E/Asx-lipo and E/D/Asx-lipo exhibited similar scavenging curves for hydroxyl radicals; these effects increased as a function of liposomal Asx concentration (Fig. 2). Taken together, these results suggest that there was little influence of the different liposomal compositions on the scavenging ability of hydroxyl radicals.

### Efficacy of liposomal Asx in an *in vitro* dry eye model on cell viability, ROS generation and up-regulation of age-related markers

**Fig. 3:**
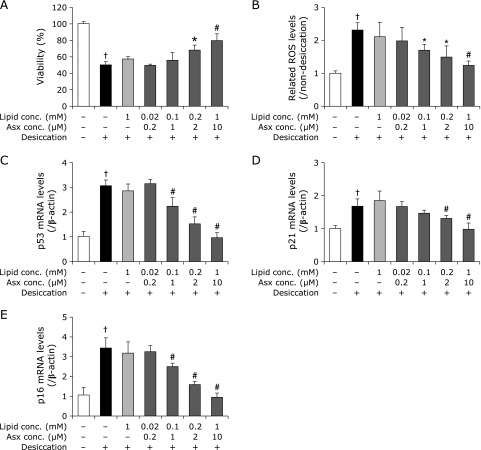
Influence of pretreatment with E/Asx-lipo or E-lipo on each marker in the *in vitro* dry eye model. Results show the effects of pretreatment with E/Asx-lipo or E-lipo on cell viability (A), related intracellular ROS levels (B), mRNA levels of p53 (C), p21 (D) and p16 (E). Data are expressed as mean ± SD (*n* = 3–5). *p<0.05, ^#^*p*<0.01 vs non-pretreatment with liposomes with desiccation as the negative control, ^†^*p*<0.01 vs non-pretreatment with liposomes without desiccation as the control.

Based on the results shown in Fig. 1, we evaluated the efficacies of each liposomal formulation on cell viability, intracellular ROS generation and up-regulation of age-related markers after 2 h of desiccation treatment. Pretreatment with E-lipo as a vehicle showed no influence on cell viability, intracellular ROS generation, or up-regulation of age-related markers (Fig. 3). In contrast, pretreatment with E/Asx-lipo improved cell viability at Asx concentrations >2 µM, and inhibited increase in intracellular ROS at concentrations >1 µM, with both effects showing a dose-dependence (Fig. 3A and B). Pretreatment with E/Asx-lipo showed significant suppression of up-regulation of age-related markers at Asx concentrations >1 µM for p53 and p16, and >2 µM for p21, compared with results obtained for the negative control. These phenomena were also dose-dependent (Fig. 3C–E). Notably, pretreatment with 10 µM E/Asx-lipo showed almost complete improvement in up-regulation of age-related markers.

### Efficacy of positively charged liposomal Asx against desiccation treatment

**Fig. 4:**
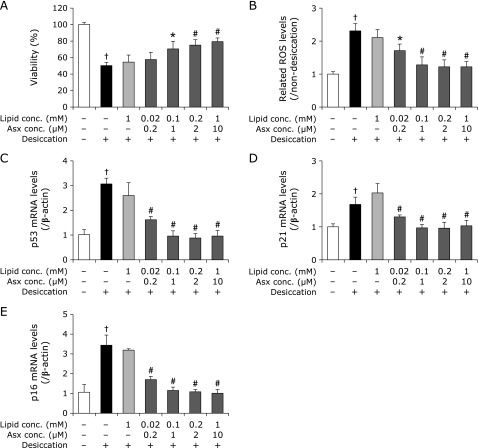
Influence of pretreatment with E/D/Asx-lipo or E/D-lipo on each marker in the *in vitro* dry eye model. Results show the effects of pretreatment with E/D/Asx-lipo or E/D-lipo on cell viability (A), related intracellular ROS levels (B), mRNA levels of p53 (C), p21 (D) and p16 (E). Data are expressed as mean ± SD (*n* = 3–5). *p<0.05, ^#^*p*<0.01 vs non-pretreatment with liposomes with desiccation as the negative control, ^†^*p*<0.01 vs non-pretreatment with liposomes without desiccation as the control.

Next, we pretreated HCECs with positively charged liposomal Asx in the *in vitro* dry eye model, and compared the results with those of E/Asx-lipo, which exhibits a neutral charge. Compared with negative control, pretreatment with E/D-lipo as a vehicle showed no effects on cell viability, intracellular ROS generation, or up-regulation of age-related markers (Fig. 4). These results were similar to those of E-lipo described above. On the other hand, pretreatment with E/D/Asx-lipo significantly suppressed up-regulation of intracellular increases in ROS (Fig. 4B), as well as all age-related markers at Asx concentrations >0.2 µM (Fig. 4C–E), and also improved cell viability at concentrations >1 µM (Fig. 4A). Moreover, efficacy toward intracellular ROS generation and up-regulation of age-related markers was demonstrated with pretreatment even at low Asx concentration, e.g., 0.2 µM. These results suggest that positively-charged liposomes containing cationic lipids, such as DOTAP, are superior to neutral ones for efficiently exerting the antioxidative effects of Asx.

### Comparison of exposure levels between E/Asx-lipo and E/D/Asx-lipo

**Fig. 5:**
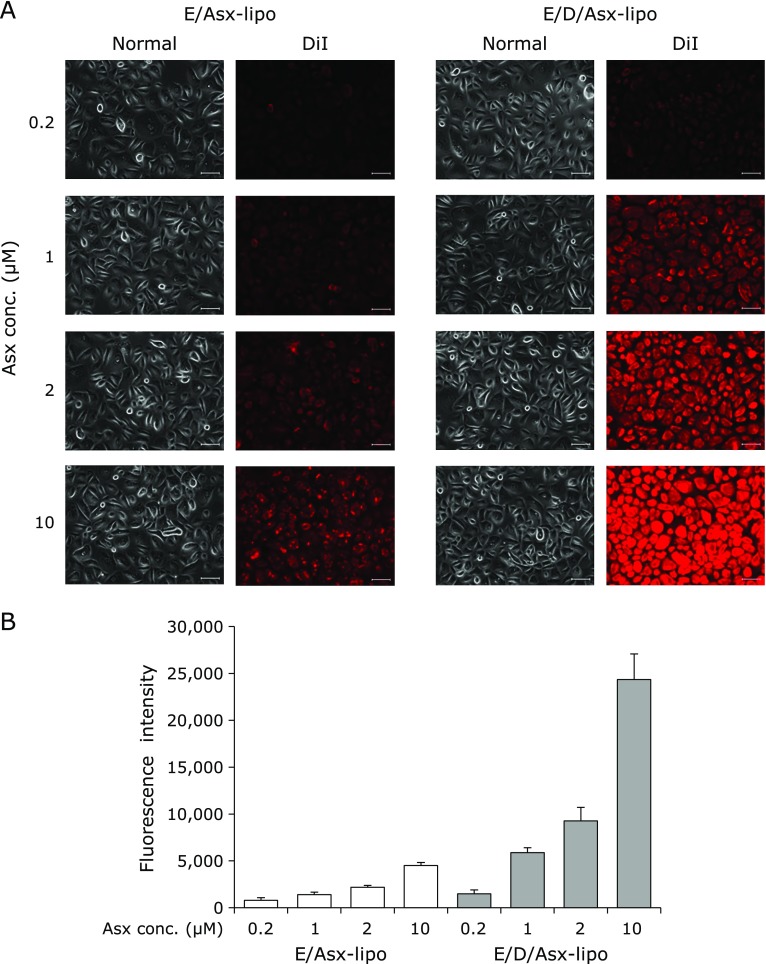
Exposure levels after treatment of E/Asx-lipo or E/D/Asx-lipo labeled with fluorescent dye DiI. Fig. 5A shows representative normal and fluorescence photographs of HCECs after 1-h treatment with E/Asx-lipo or E/D/Asx-lipo labeled with DiI. Scale bars = 50 µm. (B) Fluorescence intensities of cell lysates following 1-h treatment with E/Asx-lipo and E/D/Asx-lipo labeled with DiI. Data are expressed as mean ± SD (*n* = 3).

Greater efficacy was observed following pretreatment with positively-charged liposomes compared with neutral liposomes. As it was suspected that these differences would be induced by liposomal affinity to HCECs, we examined the exposure levels of HCECs following treatment with E/Asx-lipo and E/D/Asx-lipo labeled with the fluorescent dye DiI. Fluorescence signals of fluorescently-labeled E/Asx-lipo and E/D/Asx-lipo treated HCECs were both found to increase for 1 h post-treatment in a dose-dependent fashion, while exposure levels of HCECs were greater using liposomes comprised of EPC and DOTAP than liposomes comprised of EPC (Fig. 5A). For example, the fluorescence intensity following treatment with 10 µM E/Asx-lipo was 4510, while treatment with 10 µM E/D/Asx-lipo showed a fluorescence intensity of 24,350 (Fig. 5B). The fluorescence intensity of cells treated with 10 µM E/D/Asx-lipo was about 5 times greater than that of 10 µM E/Asx-lipo-treated cells. These results suggest that positively-charged liposomes exhibit high cellular affinity.

## Discussion

Oxidative stress caused by excessive evaporation and hyperosmolarity of tears is an important factor in the development of dry eye. In this study, we confirmed increases in intracellular ROS generation induced by desiccation stress (Fig. 1B). Our results are consistent with several reports that showed that oxidative stress is induced by desiccative stress caused by enhancement of tear evaporation and hyperosmolarity, which in turn leads to dry eye disease.^(6,8–10)^ Further, we confirmed the up-regulation of age-related markers, such as p53 and p21, as a function of increasing intracellular ROS (Fig. 1C and D). Our results are also consistent with a number of reports that demonstrated that the p53/p21 pathway is stimulated by ROS.^(29–31)^ Up-regulation of p53 and p21 is known to induce apoptosis, cell cycle arrest and senescence-associated secretory phenotype (SASP) in several cell types,^(16,29–31)^ and may contribute to the development of dry eye. We unexpectedly found significant up-regulation of p16 in this study, which, to our knowledge, is the first time this has been reported. Up-regulation of p16 caused by oxidative stress is not a well-known phenomenon. Some reports showed that p38 mitogen-activated protein kinase (MAPK) and ROS generation induced activation of p16,^(32,33)^ while other reports suggested that hyperosmolarity caused stimulation of the p38/MAPK pathway both *in vitro* and *in vivo*.^(34–36)^ Based on these reports, up-regulation of p16 may be caused by hyperosmolarity of a thin layer of medium on HCECs, which was evaporated rapidly during desiccation treatment. Although it is unknown whether oxidative stress causes up-regulation of p16 directly or indirectly, our results nevertheless suggest that oxidative stress may contribute to the up-regulation of p16, which may be an important factor in the development of dry eye.

We applied Asx-encapsulated liposomes to an *in vitro* dry eye model and confirmed inhibition of decreases in cell viability and increases in both intracellular ROS production and age-related markers (Fig. 3 and 5). These effects were considered to be due to the antioxidative ability of Asx, e.g., hydroxyl radical scavenging (Fig. 2). In fact, pretreatment with 10 µM E/Asx-lipo or 2 µM E/D/Asx-lipo reduced almost 80% of the increase in intracellular ROS production induced by desiccation treatment, while pretreatment with 10 µM E/Asx-lipo or 1 µM E/D/Asx-lipo completely inhibited the up-regulation of age-related markers (Fig. 3 and 5). These results suggest that eye drops comprised of liposomal Asx dispersed in aqueous solution may be effective as a treatment for dry eye. Further, slightly-cationic Asx liposomes demonstrated greater efficacy than neutral Asx liposomes due to the higher cellular affinity of the former (Fig. 5). We also calculated the cellular uptake ratio of fluorescently-labeled liposomes after 1 h of treatment. Treatment with 10 µM E/Asx-lipo resulted in an uptake ratio of 0.35% (relative to the amount of Asx in the medium). On the other hand, treatment with 10 µM E/D/Asx-lipo resulted in an uptake ratio of 2.0%. Eye drops for topical applications typically exhibit low bioavailability due to the drainage system associated with tear flow, and typical retention time of eye drop therapeutics on the ocular surface is known to be less than 4 min. High affinity liposomes comprised of cationic lipids should be able to facilitate new applications for drugs, such as Asx, that are difficult to prepare as eye drops due to high hydrophobicity. Such applications could further improve the bioavailability of drugs prepared for administration as eye drops.

Furthermore, it is reported that astaxanthin-treatment could prevent aging-related hyposalivation caused by dysfunction of submandibular glands, through decreasing in inflammatory cells and increasing in aquaporin-5 positive cells.^(37)^ The liposomal astaxanthin we have developed in this study not only inhibits the generation of ROS in corneal epithelium and will be expected to improve dysfunction of lacrimal glands associated with the aging process, and can be beneficial in the treatment of dry eye.

In conclusion, we confirmed the up-regulation of age-related markers, especially p16, induced by intracellular ROS in an *in vitro* dry eye model, and demonstrated that high affinity liposomal Asx could be a potential candidate for the treatment of dry eye.

## Figures and Tables

**Table 1:** List of primer sequences for real-time PCR

Gene	Forward	Reverse
p53	TAACAGTTCCTGCATGGGCGGC	AGGACAGGCACAAACACGCACC
p21	GTTCCTTGTGGAGCCGGAGC	GGTACAAGACAGTGACAGGTC
p16	GGCACCAGAGGCAGTAACCA	CCTACGCATGCCTGCTTCTACA
β-actin	CACTCTTCCAGCCTTCCTTCC	CGTACTGGTCTTTGCGGATGTC

**Table 2:** Physicochemical characteristics of each liposomal preparation

	E-lipo	E/Asx-lipo	E/D-lipo	E/D/Asx-lipo
Theoretical Asx concentration in liposomes (µM)	—	200	—	200
EPC concentration in liposomes (mM)	200	200	180	180
DOTAP concentration in liposomes (mM)	—	—	20	20
Particle diameter (nm)	145.0 ± 28.8	119.1 ± 8.5	158.5 ± 38.1	138.4 ± 7.4
PDI	0.353 ± 0.112	0.291 ± 0.058	0.357 ± 0.051	0.297 ± 0.072
Zeta potential (mV)	−3.04 ± 0.35	−3.08 ± 0.43	6.19 ± 0.35	7.82 ± 0.78
Actual Asx concentration in liposomes (µM)	—	198.3 ± 7.3	—	198.7 ± 5.4

PDI, polydispersity index. Data are expressed as mean ± SD (*n* = 5).

## Abbreviations

Asx: astaxanthin
DDR: DNA damage response
DiI: 1,1'-dioctadecyl-3,3,3',3'-tetramethylindocarbocyanine perchlorate
DOTAP: 1,2-dioleoyloxy-3-3trimethylammonium propane chloride
E/Asx-lipo: liposomes encapsulating astaxanthin using egg phosphatidylcholine as lipids
E/D/Asx-lipo: liposomes encapsulating astaxanthin using egg phosphatidylcholine and 1,2-dioleoyloxy-3-3trimethylammonium propane chloride as lipids
E/D-lipo: liposomes using egg phosphatidylcholine and 1,2-dioleoyloxy-3-3trimethylammonium propane chloride as lipids
E-lipo: liposomes using egg phosphatidylcholine as lipids
EPC: egg phosphatidylcholine
HCECs: normal human corneal epithelial cells
ROS: reactive oxygen species
